# First molecular phylogenetic and serological insights into *Listeria monocytogenes* infection in aborted ewes in Iraq: A cross-border comparative analysis

**DOI:** 10.14202/vetworld.2025.1899-1910

**Published:** 2025-07-17

**Authors:** Luma F. M. Al-Ethafa, Ahmed Jassim Almialy, Hasanain A. J. Gharban, Isra’a M. Essa, Sattar R. S. Al-Eqabi

**Affiliations:** 1 Department of Environmental Health, College of Environmental Sciences, Al-Qasim Green University, Babylon 51013, Iraq; 2 Department of Veterinary Clinical Sciences, Faculty of Veterinary Medicine, University of Kufa, Najaf 54001, Iraq; 3 Department of Internal and Preventive Veterinary Medicine, College of Veterinary Medicine, University of Wasit, Wasit 52001, Iraq; 4 Department of Public Health, College of Veterinary Medicine, University of Basrah, Basra, Iraq; 5 Department of Veterinary Public Health, Veterinary Medicine College, University of Wasit, Wasit 52001, Iraq

**Keywords:** ovine abortion, *Listeria monocytogenes*, enzyme-linked immunosorbent assay, polymerase chain reaction, phylogenetic analysis, Iraq, Iran, *16S ribosomal RNA*

## Abstract

**Background and Aim:**

*Listeria monocytogenes* is a significant zoonotic pathogen linked to reproductive losses in livestock and serious health risks in humans. In Iraq, listeriosis remains underreported in sheep, with limited data on its molecular and epidemiological characteristics. This study aimed to (1) estimate the seroprevalence of *L. monocytogenes* in recently aborted ewes, (2) evaluate the association between seropositivity and clinical indicators, and (3) perform molecular detection and phylogenetic analysis of polymerase chain reaction (PCR)-confirmed isolates.

**Materials and Methods:**

From November 2023 to August 2024, 168 aborted ewes in Wasit Province, Iraq, were sampled for vaginal swabs and blood. Enzyme-linked immunosorbent assay (ELISA) was used for serological screening, while DNA extracted from swabs underwent PCR amplification targeting the *16S ribosomal RNA* gene. PCR-positive samples were sequenced and phylogenetically analyzed using MEGA-11 software. Clinical data were statistically correlated with seropositivity using odds ratios (OR) and relative risk (RR).

**Results:**

ELISA revealed a seroprevalence of 23.21%, with the majority of infections classified as mild. PCR confirmed *L. monocytogenes* in 3.57% of swabs. Seropositivity significantly correlated with vaginal discharge (30.08%, p = 0.0121), retained placenta, and recent abortion history. Higher infection risk was observed in ewes with no or single previous abortions (OR = 2.464; RR = 2.207) and in flocks with ≤10% abortion rates (OR = 3.729; RR = 2.731). Phylogenetic analysis of six local isolates (GenBank IDs PQ865989.1–PQ865994.1) revealed 96.43%–97.62% sequence identity with an Iranian reference strain (MT071644.1), suggesting regional transmission links.

**Conclusion:**

This study is the first to molecularly characterize ovine *L. monocytogenes* in Iraq, revealing both the prevalence of subclinical infection and cross-border phylogenetic relationships. The integration of serological and molecular diagnostics highlighted underrecognized infections and provided novel insights into the epidemiology of strains. Findings emphasize the need for broader regional surveillance, improved diagnostic protocols, and biosecurity measures in ovine reproductive health management.

## INTRODUCTION

Listeriosis is a bacterial infection that affects a wide range of species, including mammals, birds, fish, crustaceans, and insects, and it tends to occur more frequently in temperate and cooler regions [1, 2]. The causative agent is *Listeria monocytogenes*, a Gram-positive, rod-shaped bacterium initially identified in necrotic rabbit livers in Sweden and later isolated from laboratory animals [3–6]. Since 1985, *L. monocytogenes* has evolved from a relatively obscure organism into a prominent foodborne pathogen with significant public health implications [7].

The infection process of *L. monocytogenes* involves several mechanisms, such as adhesion, cellular invasion, escape from vacuoles, intracellular replication, and direct cell-to-cell transmission [8]. Its pathogenicity is further enhanced by numerous virulence factors, many of which are organized within genomic islands along the bacterial chromosome [9]. Under natural conditions, the pathogen can lead to abortion, encephalitis, and septicemia in both adult and young sheep [10]. In adult sheep, infection primarily occurs through ingestion of contaminated feed or water, while lambs may acquire the infection through contaminated ewe teats, consumption of infected milk, or congenitally [1]. Abortion typically manifests from the 12^th^ week of gestation onward and may be accompanied by retained placenta and bloody vaginal discharge [11].

Prompt diagnosis is vital for the prevention and control of listeriosis in farm animals [12]. Over time, diagnostic approaches for *L. monocytogenes* have advanced from traditional cold enrichment techniques to modern conventional and molecular methods [13]. Although culture-based methods are still widely used, they suffer from drawbacks such as low specificity and limited ability to differentiate strains [14, 15]. In contrast, serological assays like enzyme-linked immunosorbent assay (ELISA) offer advantages due to their speed, widespread availability, and high sensitivity and specificity [16, 17].

Recently, polymerase chain reaction (PCR)-based molecular diagnostics have become increasingly prominent for detecting *L. monocytogenes*, with a focus on genetic targets such as the *16S rRNA* gene. Both qualitative and quantitative PCR assays are favored for their accuracy, sensitivity, specificity, simplicity, and commercial accessibility [18–20].

Despite the zoonotic importance and economic implications of *L. monocytogenes* infections in small ruminants, particularly sheep, the epidemiology of listeriosis in Iraq remains poorly understood. Most available studies in the country are either outdated, limited to specific regions (such as Nineveh, Najaf, and Babylon), or rely solely on serological data without molecular confirmation or genetic characterization of the isolates [1–24]. These limitations hinder the understanding of circulating strains, transmission dynamics, and their phylogenetic relationships with global *Listeria* populations. Moreover, there is a critical lack of integrated studies that correlate clinical outcomes in sheep with both serological and molecular diagnostic findings. The absence of national surveillance and limited molecular sequencing data further obstructs the development of effective control measures, particularly in border areas like Wasit province that may be influenced by transboundary pathogen movement. Consequently, comprehensive studies employing a One Health framework that combines clinical, serological, and molecular approaches are essential to address these knowledge gaps and support the development of evidence-based policies.

The primary aim of this study was to provide the first integrated serological and molecular investigation of *L. monocytogenes* in aborted ewes in Iraq, with a particular focus on Wasit province. Specifically, this research sought to (1) estimate the seroprevalence of listeriosis using ELISA, (2) evaluate the relationship between seropositivity and key clinical indicators such as abortion history, vaginal discharge, and gestational timing, and (3) perform molecular detection and phylogenetic analysis of *L. monocytogenes* isolates through PCR amplification and sequencing of the *16S rRNA* gene. By comparing local isolates with global reference strains, the study also aimed to explore potential regional linkages, especially with neighboring countries such as Iran. This integrative approach aims to enhance diagnostic accuracy, elucidate infection dynamics, and contribute foundational molecular data to global databases, thereby supporting regional surveillance and disease control initiatives in sheep production systems.

## MATERIALS AND METHODS

### Ethical approval

All experimental procedures involving animals were conducted in accordance with institutional animal welfare guidelines and were approved by the Scientific Committee of the College of Veterinary Medicine, University of Wasit (Approval No. 39-21/10/2023).

### Study period and location

The study was conducted from November 2023 to August 2024. This study was conducted at various sites within Wasit Province, located in the southeastern region of Iraq (32°40'N, 45°45'E), which shares a border with Iran. The region is a key center for sheep farming, with multiple breeds raised primarily for meat, milk, and wool production. Despite their economic significance, these animals frequently face health challenges, including infectious diseases that impact reproductive performance.

### Sample collection and clinical evaluation

A total of 168 ewes that had recently aborted were selected for sampling. Two vaginal swabs were collected aseptically from each ewe. In addition, 2.5 mL of venous blood was drawn into plain gel tubes (without anticoagulant) and centrifuged at 4,000 × *g* for 5 min to separate serum, which was then aliquoted into 1.5 mL labeled Eppendorf tubes (ABDOS, India). All samples were stored at −20°C until further analysis [25].

Clinical information recorded for each ewe included abortion history, flock abortion rate in the previous year, gestational age at the time of abortion, presence and type of vaginal discharge, incidence of retained placenta, neurological signs, and maternal death.

### Serological detection of *L. monocytogenes* (ELISA)

Serological testing was performed using a commercial sheep *Listeria* antibody ELISA kit (Catalog No: SL00178Sp, Sunlong Biotech, China). All reagents and samples were equilibrated to room temperature before analysis. Optical density (OD) was measured at 450 nm using an ELISA reader (BioTek, USA). Based on the control values (positive control OD: 1.241 and negative control OD: 0.067), a cutoff OD value of ≥0.217 was used to identify seropositive samples.

### DNA extraction and PCR protocol

Genomic DNA was extracted from vaginal swabs using the Presto mini gDNA bacteria kit (Geneaid, Taiwan). DNA purity and concentration were verified using a NanoDrop spectrophotometer (Thermo Scientific, UK).

Conventional PCR was conducted using primers targeting the *16S rRNA* gene of *L. monocytogenes*:

- Forward: FHAJ (5′-AGCAAGTCCAACGAAGGGAG-3′)
- Reverse: RHAJ (5′-AGCGATTCCGGCTTCATGTA-3′).

Each 20 μL reaction mix contained ×1 premix buffer, 0.5 μM of each primer, 50 ng of template DNA, and nuclease-free water. Both positive and negative-template controls were included. Amplification was carried out in a Bio-Rad thermal cycler (Bio-Rad, USA) under the following cycling conditions:

- Initial denaturation: 94°C for 7 min
- 35 cycles of denaturation at 94°C for 45 s, annealing at 54°C for 45 s, and extension at 72°C for 45 s
- Final extension: 72°C for 10 min.

PCR products were separated using 3% agarose gel electrophoresis, stained with ethidium bromide (Biotech, Canada) at 100V and 80 mA for 90 min. The bands were visualized under ultraviolet transillumination and compared with a molecular weight marker to confirm the expected 1297 bp amplicon.

### Sequencing and phylogenetic analysis

PCR-positive samples were submitted to Macrogen (Korea) for sequencing. The resulting sequences were deposited in the National Center for Biotechnology Information (NCBI) GenBank database under accession numbers PQ865989.1–PQ865994.1. Phylogenetic trees were constructed using MEGA 11.0 software (Pennsylvania State University, USA) with the Neighbor-Joining algorithm and 1000 bootstrap replications. Sequence homology and alignment were assessed using NCBI-Basic Local Alignment Search Tool (BLAST), (NCBI, USA) [26].

### Statistical analysis

All statistical analyses were performed using GraphPad Prism version 8.0.2 (GraphPad Software Inc., San Diego, CA, USA) [27]. Descriptive statistics were presented as means ± standard error or as percentages. One-sample t-tests or Wilcoxon rank-sum tests were used for group comparisons. Associations between seropositivity and clinical variables were assessed by calculating odds ratios (OR) and relative risk (RR), with statistical significance set at p < 0.05.

## RESULTS

### Serological findings

**Figure 1:**
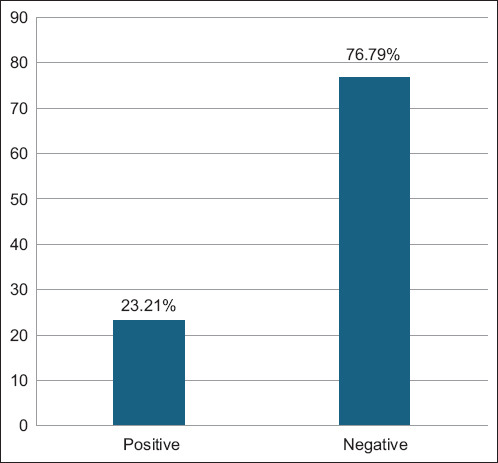
Total serological results for testing the serum samples of 168 aborted ewes by enzyme-linked immunosorbent assay.

**Figure 2:**
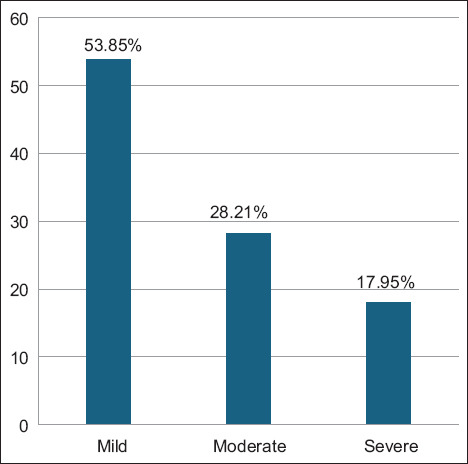
Percentage of mild, moderate, and severe infections among the 39 seropositive patients.

**Figure 3:**
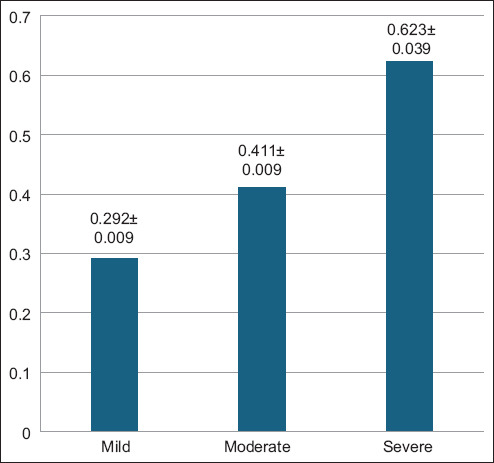
Quantitative values of mild, moderate, and severe infections among 39 seropositive patients.

ELISA screening revealed that 23.21% (39/168) of the sampled aborted ewes tested positive for *L. monocytogenes* antibodies (Figure 1). Among these seropositive animals, 53.85% were categorized as having mild infections, 28.21% as moderate, and 17.95% as severe based on OD values (Figure 2). The average OD readings were 0.292 ± 0.009 for mild, 0.411 ± 0.009 for moderate, and 0.623 ± 0.039 for severe cases (Figure 3), indicating a correlation between antibody levels and the severity of infection.

### Correlation between seropositivity and clinical parameters

A statistically significant association (p ≤ 0.0121) was found between seropositivity and the presence of vaginal discharge, which was observed in 30.08% of seropositive cases. Additional correlations were noted with retained placenta (24.49%), recent abortion within the flock (23.21%), gestational timing (23.21%), and prior abortion history in the same animal (13.46%). Notably, no seropositive animals exhibited neurological symptoms or maternal death post-abortion (Table 1).

**Table 1:** Association of seropositivity to clinical symptoms in study sheep (n = 168).

Clinical symptom	Total	Positive by serology (%)
Previous abortion (s) in each study animal	52	7 (13.46)
Percentage of abortions in the study flock in the last year	168	39 (23.21)
Association between abortion and the time of gestation	168	39 (23.21)
Incidence of vaginal discharge	123	37 (30.08)*
Incidence of placental retention	49	12 (24.49)
The occurrence of nervous signs	2	0 (0)
Mother’s death after abortion	6	0 (0)
p-value (95% confidence interval)		0.0121 (5.074–27.63)

Significant increase

* (p < 0.05)

### Abortion history and risk stratification

Listeriosis prevalence was significantly higher (p ≤ 0.0424) in ewes with no previous abortions (17.24%) and in those that had aborted once (13.33%), compared to animals with a history of two or more abortions (0%). Risk assessment indicated that ewes experiencing a single abortion had an elevated (OR = 2.464) and (RR = 2.207), underscoring their higher susceptibility to infection.

### Flock abortion rate and infection prevalence

Flock-level data from the previous year revea-led significantly increased listeriosis prevalence (p ≤ 0.0455) in flocks with ≤10% abortion rates (36.62%, OR: 3.729, RR: 2.731). In comparison, flocks with 10%–30% and ≥30% abortion rates exhibited much lower prevalence values of 14.29% (OR: 0.352, RR: 0.446) and 7.69% (OR: 0.255, RR: 0.314), respectively.

### Impact of gestational timing on infection

Although the overall prevalence of listeriosis did not significantly differ between abortions in the first (19.15%) and second (24.79%) halves of gestation (p ≤ 0.513), the RR was notably higher in the second half of pregnancy (OR: 1.675, RR: 1.292), compared to the first half (OR: 0.597, RR: 0.774).

### Vaginal discharge type and infection rate

Among seropositive animals, watery vaginal discharge was associated with the highest infection rate (34.15%, OR: 1.845, RR: 1.555), followed by mucopurulent (22.22%, OR: 0.6, RR: 0.687) and bloody discharge (21.43%, OR: 0.603, RR: 0.686), suggesting that discharge type may serve as a useful clinical indicator of *L. monocytogenes* exposure (Table 2).

**Table 2:** Distribution of seropositivity among groups according to clinical symptoms.

Clinical symptom	Total numbers	Positive (%)	OR	RR
Number of previous abortion (s) in each study animal				
0	29	5 (17.24)*	2.19	2.207****
1	15	2 (13.33)*	2.464****	0.985
2	7	0 (0)	0	0
≥3	1	0 (0)	0	0
p-value (95% CI)	-	0.0424 (6.628–21.91)	0.0001 (9.817–33.09)	0.0001 (8.693–24.65)
Percentage of abortions in a study flock in the past year				
≤10	71	26 (36.62)*	3.729****	2.731****
>10–30	84	12 (14.29)	0.352	0.446
>30	13	1 (7.69)	0.255	0.314
p-value (95% CI)	-	0.0455 (18.13–57.20)	0.0001 (34.69–63.60)	0.0001 (22.12–45.39)
Association between abortion and the time of gestation				
1^st^ half	47	9 (19.15)	0.597	0.774
2^nd^ half	121	30 (24.79)	1.675****	1.292***
p-value (95% CI)	-	0.0513 (−13.86–57.80	0.0001 (57.13–79.85)	0.001 (22.58–43.24)
Incidence of vaginal discharge				
Watery	82	28 (34.15)*	1.845****	1.555****
Mucopurulent	27	6 (22.22)	0.6	0.687
Bloody	14	3 (21.43)	0.603	0.686
p-value (95% CI)	-	0.0367 (8.229–43.64)	0.0001 (7.675–27.99)	0.0001 (26.96–58.22)

CI=Confidence interval, OR=Odds ratios, RR=Relative risk Significant increase

* (p < 0.05),

*** (p < 0.001),

**** (p < 0.0001)

### Molecular detection and phylogenetic analysis

**Figure 4:**
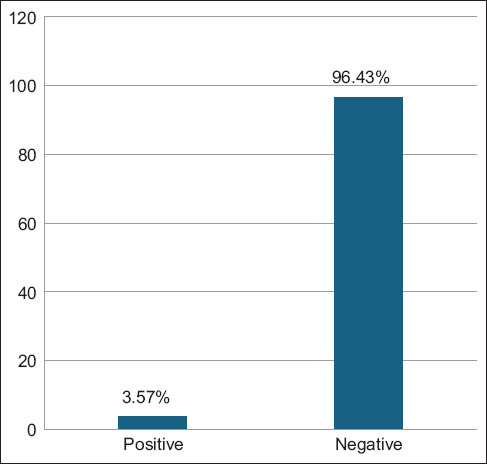
Total molecular results for testing the vaginal swab samples of 168 aborted ewes using conventional polymerase chain reaction.

**Figure 5:**
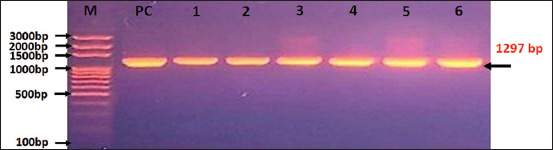
Agarose-gel electrophoresis (3%) of positive polymerase chain reaction products at 100 V and 80 mA for 90 min; in which, Lane (m): Ladder marker (100–3000 bp), Lane (PC): Positive control, and Lanes (1-6): Positive samples.

**Figure 6:**
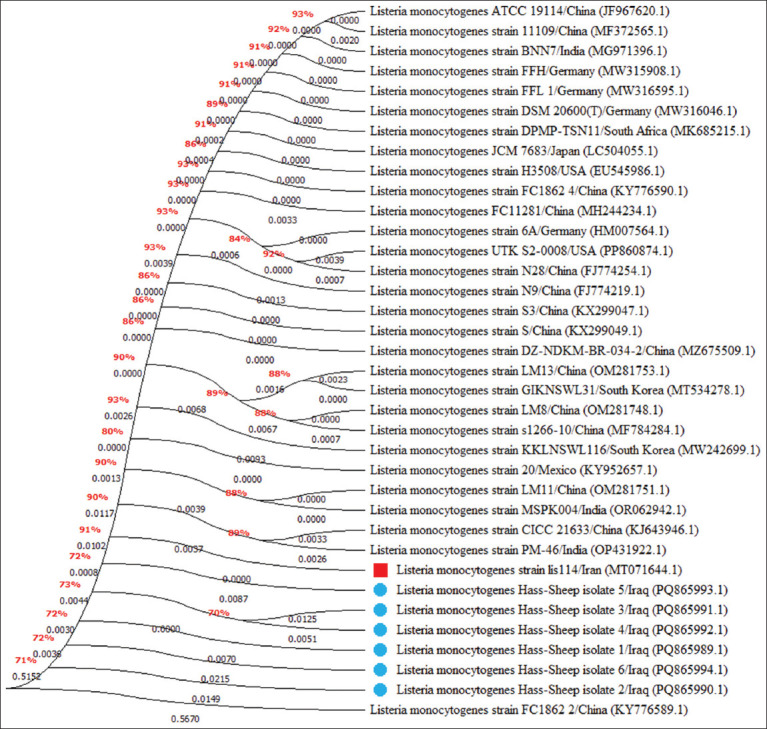
Phylogenetic tree analysis of local *Listeria monocytogenes* isolates and global National Center for Biotechnology Information-Basic Local Alignment Search Tool, *L. monocytogenes* isolates/strains.

**Figure 7:**
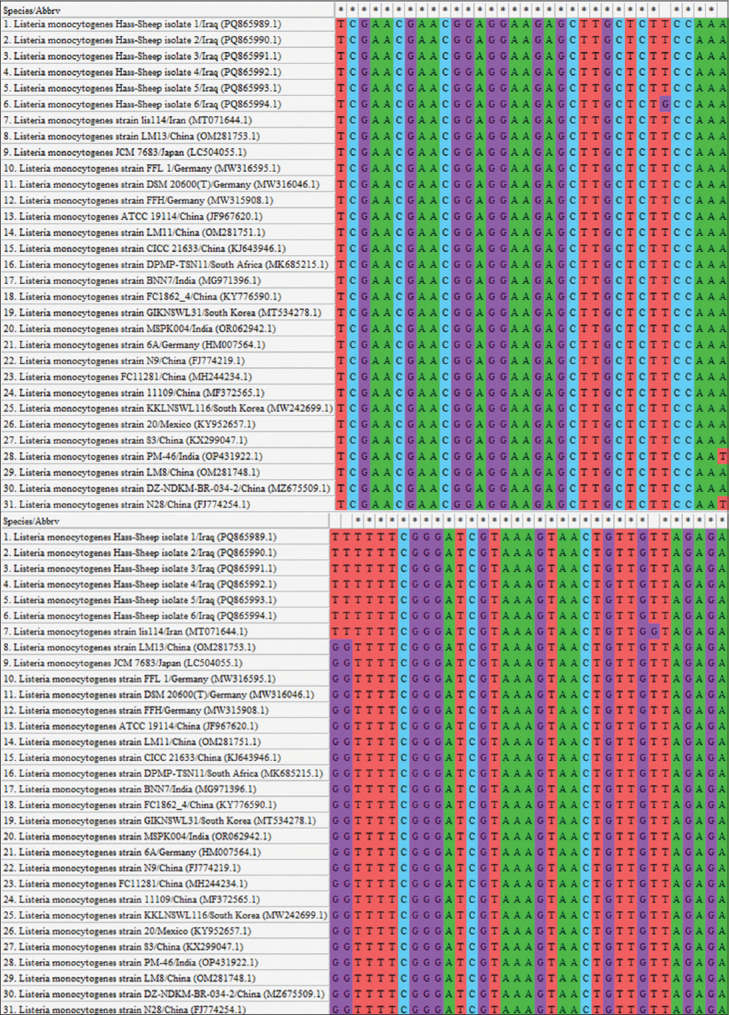
Multiple sequence alignments of the local *Listeria monocytogenes* isolates and the global National Center for Biotechnology Information-Basic Local Alignment Search Tool, *L. monocytogenes* isolates/strains.

**Figure 8:**
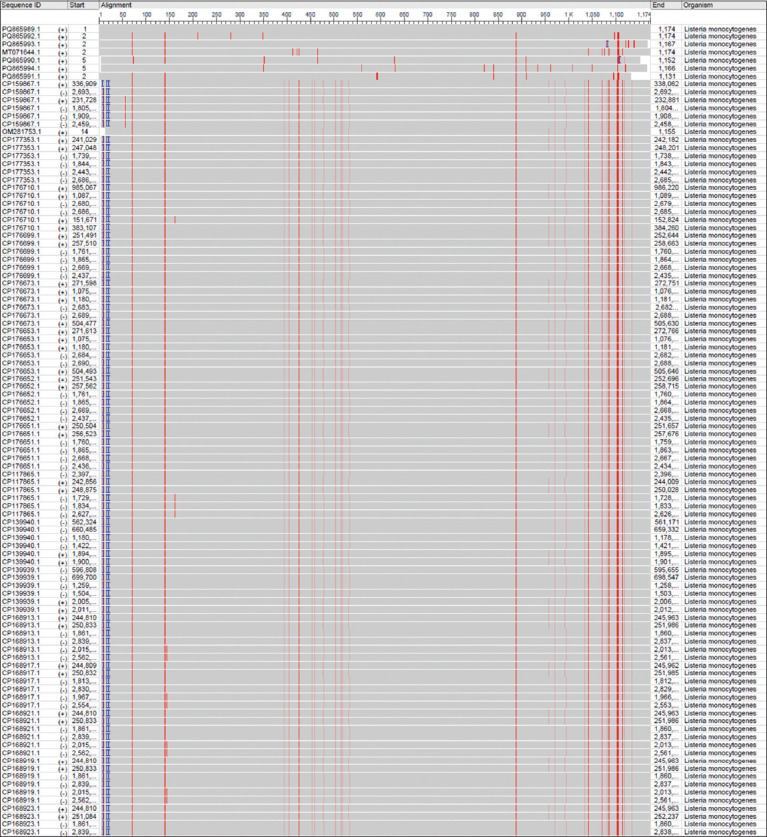
National Center for Biotechnology Information (NCBI)-Multiple sequence alignment viewer of the local *Listeria monocytogenes* isolates and the global NCBI-Basic Local Alignment Search Tool *L. monocytogenes* isolates/strains.

Out of the 168 vaginal swabs analyzed by PCR, six samples tested positive, corresponding to a molecular prevalence of 3.57% (Figures 4 and 5). These isolates were designated Hass-Sheep 1–6 and registered in the NCBI GenBank database under accession numbers PQ865989.1 to PQ865994.1. BLAST alignment and phylogenetic analysis revealed a high degree of sequence similarity (96.43%–97.62%) to the Iranian reference strain MT071644.1. The observed mutation rate ranged from 0.0016% to 0.0092%, supporting a potential epidemiological linkage across the Iraq–Iran border (Table 3 and Figures 6-8).

**Table 3:** Homology sequence identity (%) between the local *L. monocytogenes* isolates and the global NCBI-BLAST *L. monocytogenes* isolates/strains.

Local isolate		NCBI isolate			
	
Name	Accession number	Country	Source	Accession number	%
Hass–Sheep isolate 1	PQ865989.1	Iran	Sheep brain	MT071644.1	97.02
Hass–Sheep isolate 2	PQ865990.1	Iran	Sheep brain	MT071644.1	96.43
Hass–Sheep isolate 3	PQ865991.1	Iran	Sheep brain	MT071644.1	96.99
Hass–Sheep isolate 4	PQ865992.1	Iran	Sheep brain	MT071644.1	97.62
Hass–Sheep isolate 5	PQ865993.1	Iran	Sheep brain	MT071644.1	97
Hass–Sheep isolate 6	PQ865994.1	Iran	Sheep brain	MT071644.1	95.53

NCBI-BLAST=National Center for Biotechnology Information-Basic Local Alignment Search Tool, *L. monocytogenes=Listeria monocytogenes*

## DISCUSSION

### Epidemiological context and global comparison

Abortion in sheep can occur sporadically or in enzootic patterns, resulting in substantial economic losses and public health concerns. *L. monocytogenes*, a well-established foodborne pathogen, poses an elevated risk to pregnant individuals, up to 10–18 times higher than the general population, due to its ability to cross the placental barrier after ingestion of contaminated food [28, 29]. Once in the maternal bloodstream, *L. monocytogenes* can colonize the placenta, induce placentitis, and cause fetal infection, stillbirth, or early-onset neonatal listeriosis [30, 31].

In this study, a seroprevalence rate of 23.21% was observed, with the majority of cases classified as mild, underscoring the potential for subclinical infection. These findings are notably higher than those reported in prior studies from Iraq, such as Nineveh (11.6%) [32] and Babylon (12%) [33]. International comparisons reveal variability, with rates of 33.76% in India [34], 17.8% in Saudi Arabia [35], and 24.59% in Brazil [36]. In Italy, Amagliani *et al*. [37] reported that immunoglobulin G immunoassay-based positivity in asymptomatic sheep flocks rarely exceeded 10%. In Australia, *Listeria* spp. accounted for 25% of ovine abortions between 2000 and 2018, second only to *Campylobacter* spp. [38]. In Egypt, 11% of aborted ewes tested positive for *Listeria*, with the most prevalent species being *Listeria* *ivanovii* (36.8%), followed by *L. monocytogenes* (31.6%) [39].

Differences in seroprevalence across regions and studies can be attributed to variability in diagnostic methods, assay cutoff values, strain virulence, study design, and flock management practices.

### Clinical associations and risk indicators

The present study revealed significant associations between seropositivity and clinical signs, particularly vaginal discharge. Conversely, no association was observed between neurological symptoms and maternal mortality. Higher prevalence rates were noted in ewes with no history of abortion, those from flocks with ≤10% annual abortion rates, and animals that aborted during the first half of gestation. These findings suggest that even clinically mild or unremarkable cases may contribute to the silent spread of the pathogen. This aligns with observations by Abd El-Rahim *et al*. [35], who reported no visible clinical symptoms in infected sheep, indicating a possible subclinical or past exposure.

### Molecular prevalence in regional and global contexts

PCR analysis confirmed the presence of *L. monocytogenes* in 3.57% of the samples, consistent with data from Najaf province (4%) [23], but lower than that reported in Nineveh province (20.3%) [24]. Globally, molecular prevalence varies widely: 8.3% in Denmark [40], 25% in Austria [41], 6.25% in Brazil [42], 2.83% in India [43], 25% in Nigeria [44], 2.5% in Turkey [45], and between 4% and 25.4% in Egypt [46, 47], with the lowest rates reported in China (1.8%) [48]. Such variability likely results from differences in sampling strategies, animal characteristics (such as age and health status), and diagnostic methodologies, including the type and sensitivity of PCR assays used.

### Phylogenetic insights and regional transmission dynamics

Phylogenetic analysis of the *16S rRNA* gene confirmed a close relationship between local isolates and an Iranian reference strain, suggesting possible cross-border transmission or shared sources. The *16S rRNA* gene is a cornerstone in microbial identification due to its conserved and hypervariable regions, which provide high-resolution taxonomic information [49–51]. Its broad conservation across microbial taxa makes it an ideal target for classification and evolutionary studies [52, 53].

Notably, the hypervariable regions of the *16S rRNA* gene exhibit substitution rates up to 7000 times higher than those of conserved regions, enabling fine-scale phylogenetic differentiation [54–56]. This gene serves as a reliable molecular chronometer and has been instrumental in resolving taxonomic ambiguities at the strain level [57–59]. Its cost-effectiveness, speed, and accessibility have facilitated widespread adoption in both microbial ecology and biomedical research [60, 61].

By enabling detailed characterization of local strains, *16S rRNA* sequencing contributes to understanding pathogen evolution, host-pathogen interactions, and transmission dynamics. These insights are critical for monitoring regional epidemiology and informing biosecurity strategies in livestock systems [62, 63].

## CONCLUSION

This study presents the first comprehensive molecular phylogenetic and serological assessment of *L. monocytogenes* in aborted ewes in Iraq, with a focus on Wasit province. The seroprevalence was found to be 23.21%, with most cases presenting as mild subclinical infections. Molecular detection through PCR revealed a prevalence of 3.57%, and phylogenetic analysis demonstrated a close similarity (96.43%–97.62%) between local isolates and an Iranian reference strain. Significant clinical correlations were identified, particularly with vaginal discharge, retained placenta, and early gestational abortion, emphasizing the need for targeted diagnostic strategies.

The findings of this study have practical implications for livestock management and disease control. Early detection using combined serological and molecular diagnostics can help identify both clinical and subclinical infections, reducing reproductive losses and improving herd health. The generation of local molecular data and its submission to global databases will facilitate regional surveillance, risk assessment, and the formulation of control policies, particularly in cross-border livestock trade zones.

One of the key strengths of this study lies in its integrative approach, combining serological data, clinical profiling, and molecular phylogenetics for the first time in Iraqi small ruminants. The identification of relevant clinical indicators can support veterinary practitioners in early presumptive diagnosis. Moreover, the genetic sequences obtained enrich global genomic repositories and support comparative epidemiology.

However, the study had certain limitations. It was geographically confined to a single province, limiting broader generalizability. The low PCR positivity may have been influenced by sample type or bacterial shedding patterns. Furthermore, phylogenetic resolution was based solely on *16S rRNA* sequencing, which, while useful, lacks the discriminatory power of whole-genome approaches.

Future research should focus on expanding surveillance to multiple provinces and incorporating advanced molecular techniques such as multilocus sequence typing or whole-genome sequencing. Studies on environmental reservoirs, feed contamination, and antibiotic resistance profiling are also necessary to gain a holistic understanding of listeriosis transmission dynamics in Iraq.

In conclusion, this investigation reveals a substan-tial yet underrecognized burden of ovine listeriosis in Iraq, underscoring its regional epidemiological significance. The integration of serological and molecular tools enhances diagnostic capacity and provides valuable insight into pathogen transmission. The phylogenetic linkage with Iranian strains highlights the importance of regional collaboration, enhanced biosecurity, and evidence-based policy-making in controlling and preventing future outbreaks.

## DATA AVAILABILITY

All the generated data are included in the manuscript.

## AUTHORS’ CONTRIBUTIONS

LFMA: Collection of blood samples. AJA: Serological examination and statistical analysis. HAJG: Molecular examination and phylogenetic analysis of *L. monocytogenes* isolates. IME: Designed the study and extraction of DNA. SRSA: Clinical examination of study animals. All authors have read and approved the final manuscript.
